# Supplementary material for: Unexpected edge conduction in mercury telluride quantum wells under broken time-reversal symmetry
Source: Nat Commun. 2015 May 26;6:7252. doi: 10.1038/ncomms8252 (PMC4455136; doi:10.1038/ncomms8252)
Supplement: Supplementary Information — Supplementary Figures 1-5, Supplementary Notes 1-3 and Supplementary References. [file ncomms8252-s1.pdf]

Supplementary Figures

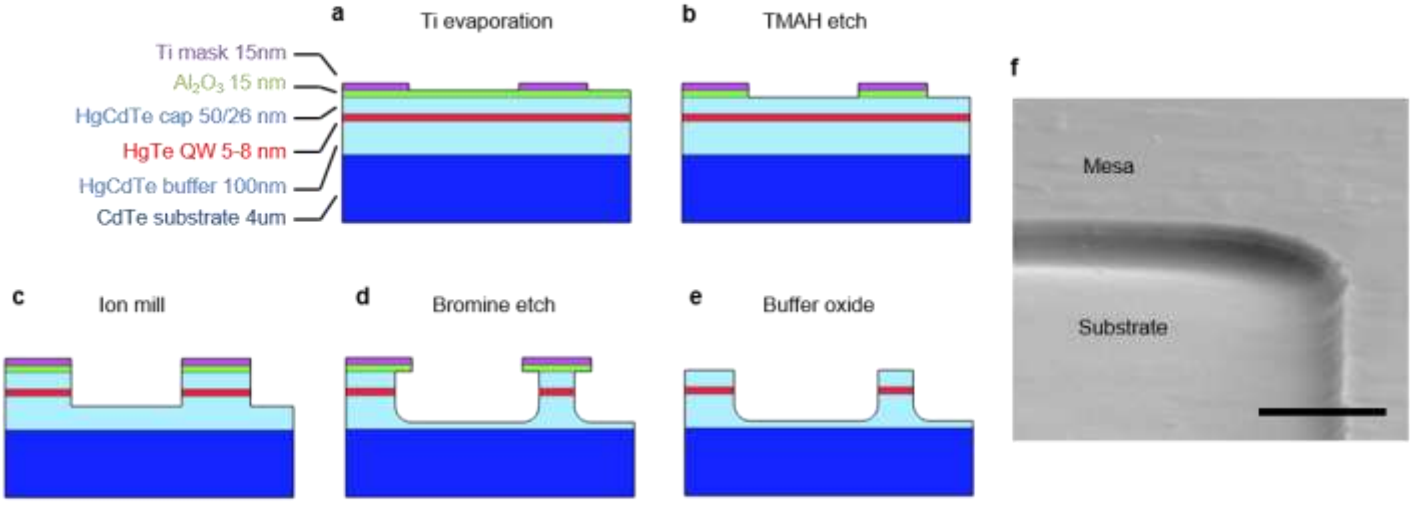

Supplementary Figure 1. Two-step etching device fabrication process. a-e, Illustration of the patterning and etching process as described in Supplementary Note 1. **f,** SEM image of a finished mesa, showing smooth clean physical edges. The scale bar is 1 µm.

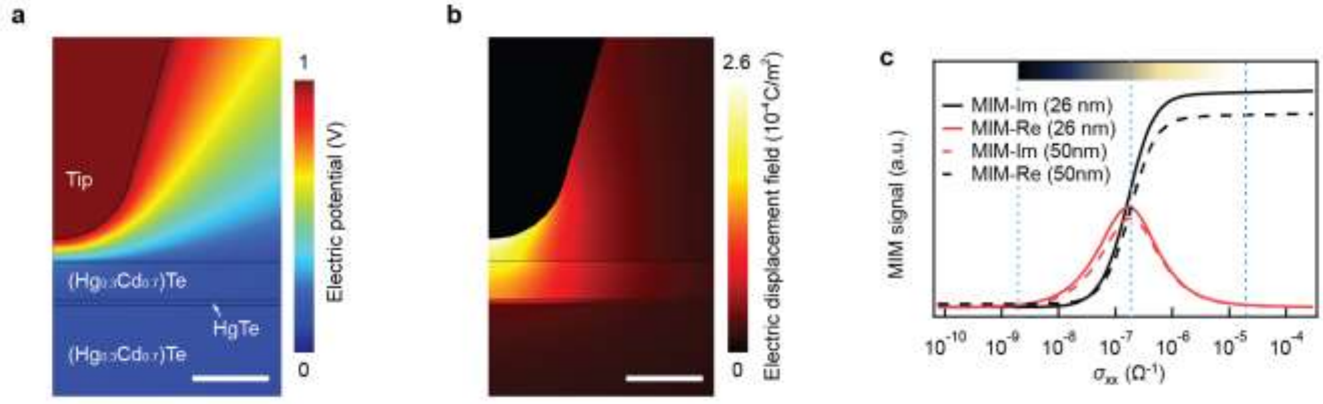

Supplementary Figure 2. Finite element analysis of MIM response. a, Electric potential profile of the axisymmetric COMSOL model for tip-sample interaction. The tip has a radius of 100 nm and is 30 nm above the top surface. The dynamic dielectric constant of HgTe, $(Hg_{0.3}Cd_{0.7})Te$ and CdTe is set to be 15.1, 8.9 and 7.2[1]. This particular profile corresponds to a QW sheet conductance of 2×10^{-7} Ω^{-1}. **b,** Electric displacement field amplitude profile of the same model. It is clear that the field is concentrated under the tip apex. **c,** MIM response curve (imaginary and real part of tip-sample admittance) for capping layer thickness of 26 (5.5 nm device) and 50 nm (7.5 nm device). The MIM signal is unnormalized here to show the minute difference: in the conductive QW limit, the 50 nm case gives a smaller total tip-sample capacitance thus a lower MIM-Im signal, as expected. In real experiments because the tip condition cannot be kept identical between different samples/cool downs, we only use the normalized MIM response curve, which is virtually identical for the 26 and 50 nm cases. The scale bars are 100 nm.

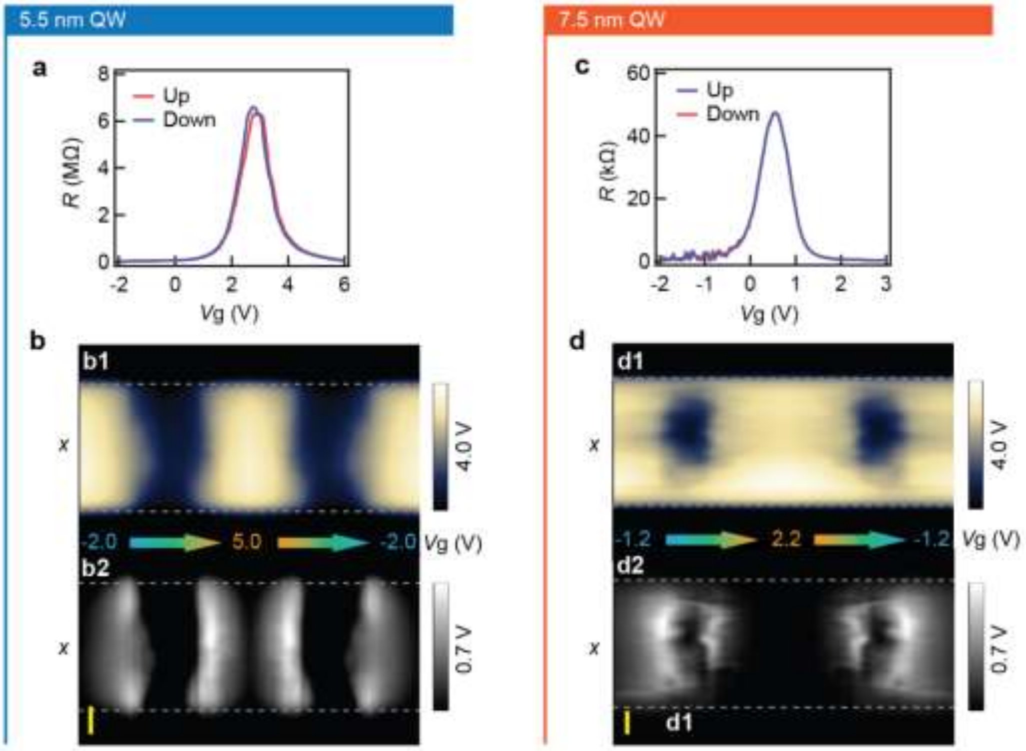

Supplementary Figure 3. Tuning the QW conductivity at zero field. a, 2-terminal resistance sweep of the 5.5 nm device showing up and down sweep, showing little hysteresis. **b,** Gate dependent MIM line-cuts with the QW being tuned from *p*-type to *n*-type and back. The perfect reflection symmetry of the MIM images show that the gate tuning is hysteresis-free and that we are probing the gate dependence of the QW without introducing any visible scanning effect. **c-d,** Same data for the 7.5 nm case, again showing perfect reflection symmetry.

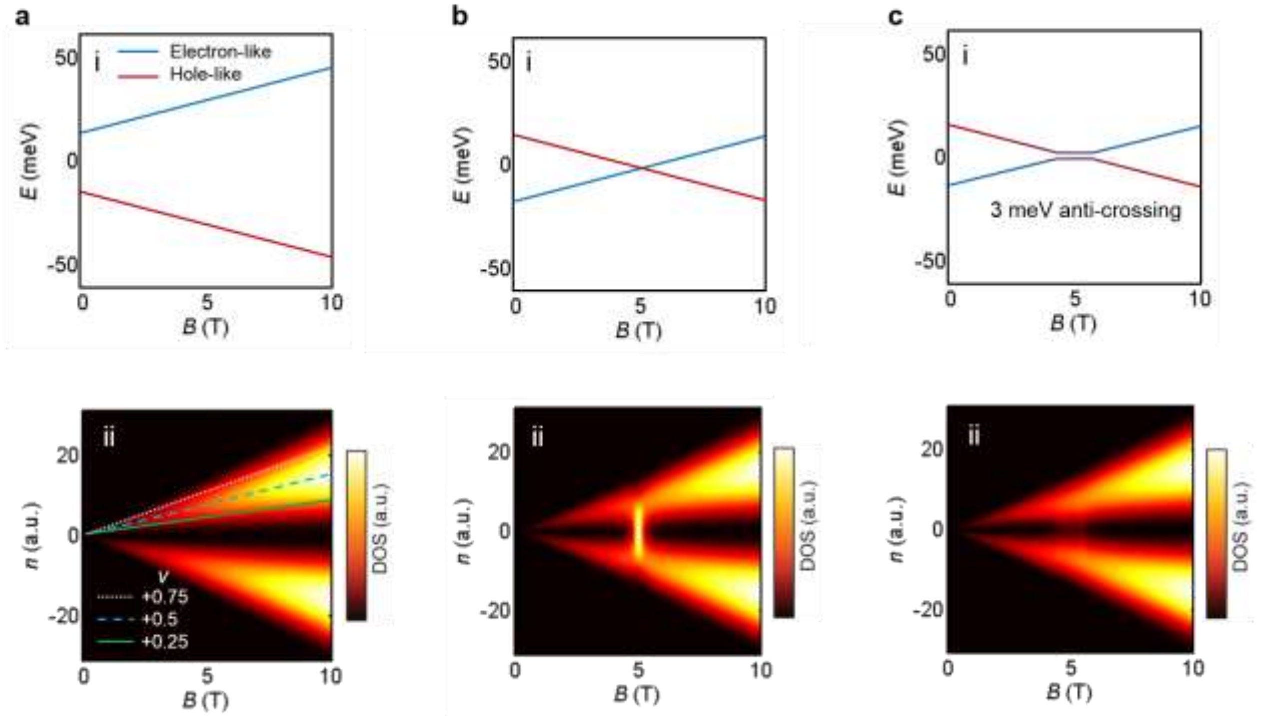

Supplementary Figure 4. Conversion from (*E*, *B*) to (*n*, *B*) representation for three toy models. Row (i) and (ii) are the LL fan charts and converted DOS plots representing **a,** a normal QW, **b,** an inverted QW with strict LL crossing and **c,** an inverted QW with avoided LL crossing. Only the lowest two LLs are included for clarity. A disorder broadening term Γ = 1.0 meV is used for the conversion. Constant filling factor corresponds to straight lines in the DOS plot, as illustrated in a(ii). The converted DOS plots are very similar because the DOS of a LL only depends on *B*. See Supplementary Note 3 for details.

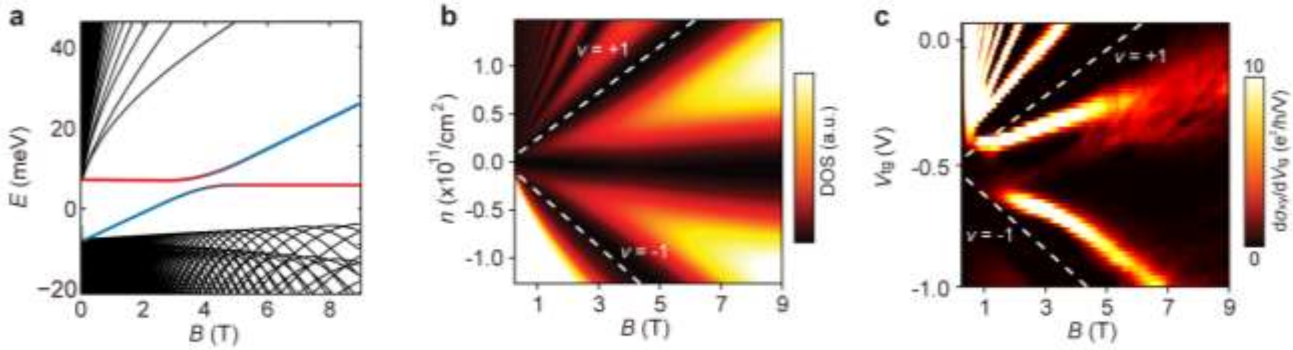

Supplementary Figure 5. Comparison between LL fan chart (*E* vs. *B*), converted DOS plot (*n* vs. *B*) and transport data from a gated 7.5 nm HgTe QW device (*Vg* vs. *B*). a, calculated LL fan chart of a 7.5 nm HgTe QW. **b,** converted DOS plot from a. **c,** first derivative of Hall conductance vs. gate voltage taken from a top-gated Hall bar device made from the same 7.5 nm QW wafer as studied in the main text. Zero derivative corresponds to plateaus in Hall conductance around integer filling factors. Densities (gate voltages) corresponding to filling factor ν = ±1 are labeled in b and c. It is obvious that gate- and field-dependent experimental data is only directly comparable to the converted DOS plot (*n* vs. *B*) thus no re-entrant behavior is expected. See Supplementary Note 3 for details.

Supplementary Notes

Supplementary Note 1. Two-step etching process for device fabrication

The samples were fabricated following a two-step etching recipe in order to produce clean physical edges and avoid artifacts resulting from dielectric or even conductive material re-deposition at the mesa edge. The temperature during the fabrication process was kept below 80°C to prevent damaging the HgTe quantum well properties[2].

A 15 nm titanium mask was used to define the mesa pattern during the etching process. To prevent the titanium from reacting with the mercury compounds, a 10 nm sacrificial layer of Al_2O_3 was first grown by low temperature (60°C) atomic layer deposition (ALD) covering the whole chip. On top of this, the titanium mask was patterned by optical lithography and e-beam evaporation (Supplementary Fig. 1a). After lift-off, the sample is put in a solution containing TMAH (Microposit -CD26 developer) for 40 seconds, which removes the aluminum oxide layer not covered by the titanium mask, as sketched in Supplementary Fig. 1b.

The mesa is now defined through two etching steps: first, we dry-etch 100 nm of material by argon ion milling. In order to remove any re-deposited material at the mesa edges (Supplementary Fig. 1c), we now chemically etch the mesa by introducing the sample in a solution of 1:1400 bromine in ethylene-glycol for one minute (Supplementary Fig. 1d). The etching rate of this process slightly varies, typically from 50 to 70 nm/min. Once the mesa is defined, the mask is removed by dipping the sample for 10 minutes in a 1:20 HF buffer oxide solution (Supplementary Fig. 1e). The resulting mesas have smooth clean physical edges (Supplementary Fig. 1f). Ohmic contacts are then deposited[2].

Supplementary Note 2. Calculation of MIM response curve and calibration of MIM-Im color scale

MIM-Im/Re is linearly proportional to the imaginary and real part of the tip-sample admittance Y_{TS} (1 V of MIM signal ~ 3.5 nS of ΔY_{TS} in this particular experiment), which can be obtained via finite element analysis. We build a realistic tip-sample model and simulate Y_{TS} in COMSOL Multiphysics 4.4 (Supplementary Fig. 2). A quasi-static model is enough because the length scale of tip-sample interaction is much smaller than the microwave wavelength. Plotting Y_{TS} as a function of QW conductivity gives the MIM response curve in Fig. 1b in main text. The difference due to different capping layer thickness is minute because the capacitance of the 30 nm vacuum gap is much larger than that of the capping layer.

To calibrate the two-tone color scale for MIM-Im, one can use an image that has sufficient variation of QW conductivity (e.g. a gate dependent scan) that covers the full MIM sensitivity window. The regime with vanishing MIM-Re and the highest MIM-Im sets the conductive limit (σ_{xx} >~2x10^{-5} Ω^{-1}); the regime with vanishing MIM-Re and the lowest MIM-Im sets the insulating limit (σ_{xx} <~2x10^{-9} Ω^{-1}); a self-consistency check is whether the regime with intermediate MIM-Im has maximum MIM-Re signal (σ_{xx} ~2x10^{-7} Ω^{-1}). One can then assign two tones to the calibrated color scale to represent the more conductive and more insulating halves of the MIM sensitivity window which makes MIM-Im images very intuitive semi-logarithmic representations of local conductivity (Supplementary Fig. 2c). Due to the presence of topographic features and stray capacitive coupling, such a calibration should not be used in a very quantitative manner; on the other hand MIM is usually used to identify the relative contrast of conductivity (as in this work) in which case an order-of-magnitude calibration is sufficient.

Supplementary Note 3. Conversion from (*E*, *B*) to (*n*, *B*) representation of LLs and the "re-entrant quantum Hall effect"

LL fan charts are obtained by calculating the energy of various LLs as a function of magnetic field. In most electronic transport experiments however, one does not have direct access to control or measure energy; instead, one often uses back and/or front gates to control the carrier density in the QW. The QW and the gate act like a parallel plate capacitor: the gate voltage linearly tunes the excess carrier density in the QW (to the 1st order, ignoring contribution from quantum capacitance); a constant gate voltage therefore corresponds to a constant amount of *excess carriers*, instead of constant *Fermi level*. Consequently, one needs to first convert the LL fan chart (*E* vs. *B*) to a DOS plot (*n* vs. *B*) before directly comparing it to gate- and field-dependent experimental data.

We now demonstrate why drastically different LL fan charts can result in very similar (*n*, *B*) DOS plots. The major reason is simply that the DOS of a single LL depends only (linearly) on *B*. Therefore for a given density and magnetic field, one can immediately calculate the LL filling factor which in turn gives the DOS, without knowing the LL fan chart at all, as long as the disorder and thermal broadening are much smaller than the minimum gap size between relevant LLs (Supplementary Fig. 4a). This picture breaks down near LL crossing points, which manifest themselves as vertical lines with high DOS connecting adjacent LLs (Supplementary Fig. 4b). If instead a moderate anti-crossing takes places, the vertical line disappears quickly and the universal form of the (*n*, *B*) DOS plot is restored (Supplementary Fig. 4c). For a more detailed example for real materials see the Supplementary Information of ref. 3.

The implication here is that if E_F is in the bulk gap at zero field, it will stay in the gap, regardless of the behavior of the LLs in the fan chart, as long as a moderate gap is maintained (as is the case for the 7.5 nm QW studied here). In a previous study[4] magnetotransport taken with a constant gate voltage was interpreted as taking a constant E_F cut in the LL fan chart (Fig. 1 therein), and a so called 're-entrant quantum Hall effect' was expected, where E_F starts in the gap, crosses multiple LLs and goes back to the gap, due to the crossing of the lowest LLs. From the discussion above we recognize that it was not the most accurate: magnetotransport taken with a constant gate voltage should correspond to a constant *density* cut in the *converted DOS plot* and no re-entrant behavior is expected, as can be seen clearly from the comparison between LL fan chart, DOS plot and transport data (Supplementary Fig. 5). In other words, when the gate voltage is kept constant, the excess charge needed for E_F to cross LLs is simply not supplied.

Supplementary References

1. Brice, J. C. In Properties of mercury cadium telluride, EMIS Datareviews Series No.3 (INSPEC, IEE, 1987) ch.2 p.29.

2. Baenninger, M. *et al.* Fabrication of samples for scanning probe experiments on quantum spin Hall effect in HgTe quantum wells. *J. Appl. Phys*. **112**, 103713 (2012).

3. Taychatanapat, T., Watanabe, K., Taniguchi, T. & Jarillo-Herrero, P. Quantum Hall effect and Landau-level crossing of Dirac fermions in trilayer graphene. *Nat. Phys.* **7,** 621–625 (2011).

4. König, M. *et al.* Quantum spin hall insulator state in HgTe quantum wells. *Science* **318,** 766-770 (2007).
